# Risk factors for invasive aspergillosis in ICU patients with COVID-19: current insights and new key elements

**DOI:** 10.1186/s13613-021-00923-4

**Published:** 2021-09-15

**Authors:** G. Montrucchio, T. Lupia, D. Lombardo, G. Stroffolini, S. Corcione, F. G. De Rosa, L. Brazzi

**Affiliations:** 1 Department of Surgical Sciences, University of Turin, 10126 Turin, Italy; 2 Department of Anaesthesia, Critical Care and Emergency - Città Della Salute E Della Scienza Hospital, Corso Dogliotti 14, 10126 Torino, Italy; 3 Unit of Infectious Diseases, Cardinal Massaia Hospital, Asti, Italy; 4 Department of Medical Sciences, University of Turin, Infectious Diseases, Città Della Salute E Della Scienza Hospital, Turin, Italy; 5 Tufts University School of Medicine, Boston, USA

**Keywords:** COVID-19, SARS-CoV-2, CAPA, COVID-19-associated pulmonary aspergillosis, Acute respiratory distress syndrome, Invasive pulmonary aspergillosis

## Abstract

Invasive pulmonary aspergillosis (IPA) has always been a challenging diagnosis and risk factors an important guide to investigate specific population, especially in Intensive Care Unit. Traditionally recognized risk factors for IPA have been haematological diseases or condition associated with severe immunosuppression, lately completed by chronic conditions (such as obstructive pulmonary disease, liver cirrhosis, chronic kidney disease and diabetes), influenza infection and Intensive Care Unit (ICU) admission. Recently, a new association with SARS-CoV2 infection, named COVID-19-associated pulmonary aspergillosis (CAPA), has been reported worldwide, even if its basic epidemiological characteristics have not been completely established yet. In this narrative review, we aimed to explore the potential risk factors for the development of CAPA and to evaluate whether previous host factors or therapeutic approaches used in the treatment of COVID-19 critically ill patients (such as mechanical ventilation, intensive care management, corticosteroids, broad-spectrum antibiotics, immunomodulatory agents) may impact this new diagnostic category. Reviewing all English-language articles published from December 2019 to December 2020, we identified 21 papers describing risk factors, concerning host comorbidities, ICU management, and COVID-19 therapies. Although limited by the quality of the available literature, data seem to confirm the role of previous host risk factors, especially respiratory diseases. However, the attention is shifting from patients’ related risk factors to factors characterizing the hospital and intensive care course, deeply influenced by specific features of COVID treatment itself. Prolonged invasive or non-invasive respiratory support, as well as the impact of corticosteroids and/or immunobiological therapies seem to play a pivotal role. ICU setting related factors, such as environmental factors, isolation conditions, ventilation systems, building renovation works, and temporal spread with respect to pandemic waves, need to be considered. Large, prospective studies based on new risk factors specific for CAPA are warranted to guide surveillance and decision of when and how to treat this particular population.

## Background

Traditionally, recognized risk factors for invasive pulmonary aspergillosis (IPA) have been underlying haematological diseases or condition associated with severe immunosuppression (e.g., neutropenia, hematopoietic stem-cell-transplant, chemotherapy, TNF-α inhibitors or prolonged corticosteroid therapy) and, lately, other factors have been added to the list (e.g., severe inherited immunodeficiencies and low CD4 count).

Other favourable environments for IPA include chronic obstructive pulmonary disease (COPD), liver cirrhosis, systemic connective tissue diseases, chronic kidney disease (CKD) or renal replacement therapy (RRT), influenza infection [1], diabetes mellitus (DM) and advanced solid cancer.

Less defined risk factors include non-traditional and emerging patient groups potentially exposed to invasive pulmonary aspergillosis risk such as critically ill patients admitted to Intensive Care Unit (ICU) [2].

Unfortunately, in ICU, the diagnosis of IPA remains troublesome due to the fact that proven diagnosis of IPA requires a positive pulmonary biopsy for *Aspergillus* spp. For that reason, is often a late or post-mortem diagnosis. Additionally, clinical and radiological signs of IPA in ICU patients are shared by many pulmonary aspergillosis mimickers (e.g., bacterial infections, water overload, atelectasis) which may delay clinical suspicion. Finally, it is known that the IPA diagnosis requires adequate training to perform samples of the lower respiratory tract in critically ill patients for direct (e.g., culture) or indirect (e.g., galactomannan) fungal microbiological confirmation.

Blot and colleagues [3] proposed a clinical diagnostic algorithm to identify patients not reflecting classic risk factors as defined by the European Organization for Research and Treatment of Cancer/Mycosis Study Group Education and Research Consortium (EORTC/MSGERC) [4], but showing the simultaneous presence of clinical and radiological criteria combined with risk factors and positive cultures for *Aspergillus* spp., and therefore fulfilling the criteria for putative invasive pulmonary aspergillosis (PIPA). Recently, IPA has emerged as a frequently reported complication in ICU patients with severe influenza and mechanical ventilation, defining influenza-associated aspergillosis (IAPA) has a new significant condition with a relevant impact on mortality [1, 5].

The increasingly widespread SARS-CoV-2 infection [6] is now known to be associated with immune dysregulation, affecting both T helper 2 (Th2 and Th1) responses [7, 8]. Although a direct immunomodulatory effect on host antifungal defence has yet not been demonstrated [9], it is believed likely that patients affected by COVID-19, or the treatment reserved for them, can facilitate the onset of *Aspergillus* spp. infections or make them particularly important to identify early.

While it is true that the definition of IPA has been further revised in the context of the current pandemic, coining the acronym CAPA (COVID-19 associated pulmonary aspergillosis) [10], there is still missing evidence about the relative incidence of risk factors in the context of CAPA patient population. Nonetheless, in patients with COVID-19-related ARDS and severe secondary infection mortality was reported to be 16% and 25% higher than that of patients without evidence of aspergillosis [11, 12].

We undertook the present narrative review of the literature to explore the potential risk factors for the development of invasive pulmonary aspergillosis in COVID-19 patients and to evaluate whether the therapeutic approaches used in the treatment of critically ill patients with severe SARS-CoV-2 pneumonia may impact this new diagnostic category.

## Search strategy and results

**Fig. 1:**
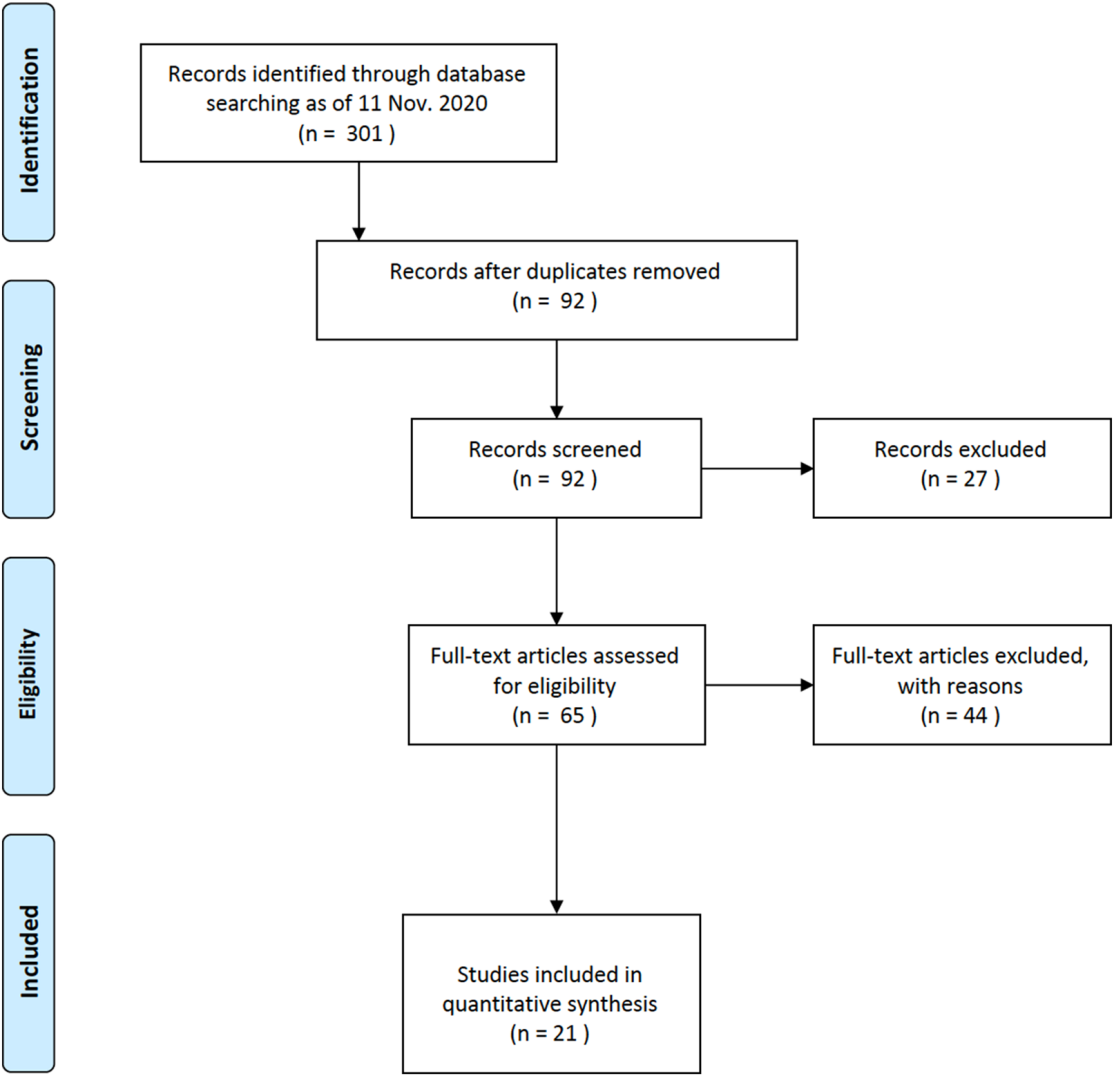
Flow diagram of studies selection

All English-language research articles published from December 1st, 2019, to December 1st, 2020 and retrieved in Europe PubMed Central in Europe PubMed Central, Google scholar, Cochrane and registries (clinicaltrial.gov) using MeSH terms using MeSH terms: “COVID-19” [All Fields] OR “SARS-CoV-2” [All Fields] AND {“Aspergillosis” [MeSH Terms]; “COVID-19” [All Fields] OR “SARS-CoV-2 [All Fields] AND “Aspergillus” [MeSH Terms]; “COVID-19” [All Fields] OR “SARS-CoV-2 [All Fields] AND “ CAPA” [MeSH Terms] were screened according to the eligibility criteria revised by GM, DL, TL and GS. References of included original studies were also screened. Case-series were considered if included ≥ 2 patients. Reviews and case reports were excluded (Fig. 1).

## Results

Applying the search criteria described above, 301 potentially eligible items were identified. Two hundred and nine were excluded as they were simultaneously identified in different databases. Of the 92 remaining articles, 27 were excluded at the first screening because they were not relevant (animal studies, in vitro studies, others). Of the 65 articles evaluated, 44 were further excluded because they did not follow the inclusion criteria (case reports, opinions, letters to the editor, others).

The remaining 21 papers identified the following as main risk factors for the development of invasive pulmonary aspergillosis in COVID-19 patients: (1) host comorbidities (respiratory, cardiovascular and hepatic disease; diabetes; renal failure (including haemodialysis and/or renal replacement therapy); (2) aspects related to ICU management (time to diagnosis; use of respiratory support; intercurrent infections; previous antibiotics and antifungal treatment; and (3) COVID-19 immunomodulating therapies (Tables 1 and 2).

**Table 1:** Summary of studies that reported pulmonary aspergillosis among patients with COVID-19

Reference	Country	Patients Included (patients with IA/ total of patients)	ICU Patients	MV Patients	IA: diagnosis from ICU admission (days)	Criteria / Definitions used	BAL GM +	Serum GM +
Alanio	France	9/27	9	9	NA	CAPA definition (Verweij) AspICU (Blot) Modified AspICU (Schauwvlieghe) EORTC/MSG	1/7	1/9
Bartoletti	Italy	30/108	30	30	4 (2 to 8)	CAPA definition (Verweij) AspICU (Blot) Modified AspICU (Schauwvlieghe)	30/30	1/30
Borman	United Kingdom	61	61	61	NA	NA	5/61	
Dupont	France	19/106 (17,9%)	19	18	10 (4 to 23)	AspICU (Blot)	5/9 tested	NA
Falces-Romero	Spain	10	7	7	NA	AspICU (Blot) EORTC/MSG	2/2 tested	1/2 tested
Flikweert	Netherlands	7	7	7	19,5 (1 to 24)	custom (symptoms and GM + on BAL/serum OR positive BAL cultures)	6/7 (GM + on fluids, distinction between BAL or Serum NA)	NA
Gangneux	France	7/45	NA	7	NA	CAPA definition (Verweij) AspICU (Blot) Modified AspICU (Schauwvlieghe)	NA	2/7
Helleberg	Denmark	2/8	2	2	5 and 1 days	NA	1/1 tested	1/2
Koehler	Germany	5/19	5	5	NA	CAPA definition (Verweij) AspICU (Blot) Modified AspICU (Schauwvlieghe)	3/3 tested	2/5
Lamoth	Switzerland	3/80	NA	3	7 (3 to 8)	CAPA definition (Verweij) AspICU (Blot) Modified AspICU (Schauwvlieghe) EORTC/MSG	NA	1/3
Marr	USA / Spain	20	20	20	9,5 (-1 to 24)	custom (positive respiratoy fluids cultures OR GM + on serum/BAL or BDG serum)	0/1 tested	4/16 tested
Mitaka	USA	4/7	NA	4	6,8 (1 to 14)	AspICU (Blot)	NA	1/3 tested
Nasir	Pakistan	5/23	5	5	8	custom (clinical parameters, radiological findings and mycological data)	NA	0/9 tested
Rothe	Germany	2/140	NA	NA	NA	NA	NA	NA
Rutsaert	Belgium	7/34	7	7	8 (5 to 13)	CAPA definition (Verweij) AspICU (Blot) Modified AspICU (Schauwvlieghe) EORTC/MSG	5/6	1/6
Van Biesen	Netherlands	9/42	9	9	3 (1 to 19)	AspICU (Blot)	9 tested	NA
Van Arkel	Netherlands	6/31	6	6	5 (3 to 14)	CAPA definition (Verweij) AspICU (Blot) Modified AspICU (Schauwvlieghe)	2/3 tested	0/3 tested
Wang	China	8/104	8	4	19	custom (positive BAL or sputum cultures)	NA	NA
White	United Kingdom	25/135	NA	25	NA	1,2 + modified IAPA guidelines to accepted NBL GM-EIA positivity in place of testing BAL	NA	1/2 tested
Ichai*	France	6/26	6	6	NC	NC	NC	NC
Zuo*	China	6/30	6	6	NC	NC	NC	NC
Overall		239 patients			median 8 days from ICU admission (median 6,8 days of MV)		60/133 tested	16/97 tested

*BAL* broncho-alveolar lavage, *EIA* enzyme immunoassays, *GM* galactomannan, *GMI* galactomannan index, *IA* invasive aspergillosis, *ICU* intensive care unit, *MV* mechanical ventilation, *NA* not applicable, *NC* not classifiable, *NBL* non-directed bronchial lavage, *SV* spontaneous ventilation

GM + if GMI on BAL > 1,0 or serum > 0,5

^*^article was not used in "Results" but only in "Discussion" modified from [13]

**Table 2:** Summary of studies and risk factors

References	Patients Included (patients with IA/ total of patients)	Respiratory diseases	Smoking	Cardiovascular disease	Hepatic disease	Diabetes	Chronic Kidney Disease	IA: diagnosis from ICU admission (days)	Type of respiratory support	Steroids	Other imunomodulatory therapies	Intercurrent infections	Previous antimicrobial treatment
Alanio	9/27	2/9	NA	7/9	NA	3/9	NA	NA	MV	6/9	NA	NA	NA
Bartoletti	30/108	4/30	NA	19/30	NA	5/30	6/30	4 (2 to 8)	MV	18/30	22/30	NA	NA
Borman	61	NA	NA	NA	NA	NA	NA	NA	MV	NA	NA	NA	NA
Dupont	19/106	6/19	NA	8/19	1/19	7/19	2/19	10 (4 to 23)	MV, SV	7/19	NA	19/19	NA
Falces-Romero	10	4/10	NA	1/10	NA	5/10	NA	NA	MV	10/10	4/10	NA	NA
Flikweert	7	NA	NA	NA	NA	NA	NA	19,5 (1 to 24)	MV	4/7	NA	NA	NA
Gangneux	7/45	0/7	1/7	5/7	NA	2/7	1/7	NA	MV	NA	NA	NA	NA
Helleberg	2/8	1/2	2/2	1/2	NA	NA	NA	5 and 1 days	MV	1/2	0/2	None documented	2/2
Koehler	5/19	2/5	2/5	2/5	1/5	1/5	NA	NA	MV	3/5	NA	NA	NA
Lamoth	3/80	2/3	NA	2/3	NA	1/3	NA	7 (3 to 8)	MV	NA	3/3	NA	3/3
Marr	20	7/20	NA	12/20	1/20	6/20	3/20	9,5 (-1 to 24)	MV	4/20	6/20	NA	NA
Mitaka	4/7	1/4	NA	1/4	0/4	1/4	1/4	6,8 (1 to 14)	MV	4/4	1/4	NA	4/4
Nasir	5/23	NA	NA	5/9	NA	8/9	NA	8	MV	5/5	3/5	5/5	5/5
Rothe	2/140	NA	NA	NA	NA	NA	NA	NA	MV, SV	NA	NA	Yes (number not specified)	2/2
Rutsaert	7/34	NA	NA	3/7	NA	3/7	1/7	8 (5 to 13)	MV	1/7	NA	NA	NA
Van Biesen	9/42	4/9	NA	3/9	NA	1/9	NA	3 (1 to 19)	MV	1/9	NA	NA	NA
Van Arkel	6/31	3/6	NA	1/6	NA	NA	NA	5 (3 to 14)	MV	2/6	0/6	NA	NA
Wang	8/104	2/8	2/8	8/8	NA	2/8	2/8	19	MV, SV	6/8	NA	NA	6/8
White	25/135	12/25	NA	10/25	1/25	6/25	1/25	NA	MV	16/25	NA	Yes	Yes (number not specified)
Overall													
Studies considering the risk factor/total of studies		14/21	4/21	16/21	5/21	14/21	8/21	12/21		15/21	8/21	3/21	7/21
Patients with the considered risk factor/ total of patients in studies	239 with IA	50/157	7/22	88/173	4/73	51/165	17/120	median 8 days from ICU admission		88/166	39/80	NA	NA

*BAL* broncho-alveolar lavage, *EIA* enzyme immunoassays, *GM* galactomannan, *GMI* galactomannan index, *IA* invasive aspergillosis, *ICU* intensive care unit, *MV* mechanical ventilation, *NA* not applicable, *NC* not classifiable, *NBL* non-directed bronchial lavage, *SV* spontaneous ventilation

Although not mentioned in the 21 selected studies, environmental and logistic factors were considered by some authors for their relevance, are briefly discussed in this review.

### Host comorbidities

#### Respiratory disease

Several authors described the presence of respiratory comorbidities in populations of patients admitted to intensive care for COVID-19 and colonized by Aspergillus spp. These include chronic obstructive pulmonary disease (COPD) [10, 11, 13–15], asthma [15–19] and smoking [20, 21].

#### Cardiovascular disease

In the CAPA context, arterial hypertension [10, 11, 13, 19, 22–25] and coronary heart disease [11, 14, 19, 22] have been frequently reported while Van Arkel et al. [15] reported on one patient with underlying cardiomyopathy.

#### Hepatic disease

None of the identified studies identified liver disease as a risk factor for CAPA while some authors [10, 12, 26, 27] hypothesized a possible role of chronic hepatitis B infection on the development of IPA / CAPA. Falces-Romero et al. [14], despite the lack of a confirmed diagnosis of aspergillosis according to the MSG criteria, suggested that other indirect causes of liver failure, such as steatosis or obesity, could be considered as risk factors.

#### Diabetes

Diabetes (not type 1) has been described as a common condition in patients with SARS-CoV-2 infection complicated by CAPA [10–12, 14, 16, 19, 22, 24–26, 28–33].

#### Renal failure

Renal disease has been reported as a pre-existing factor [12, 19, 22, 24–26, 28–30, 33] for COVID-19 infection with no ascertained difference in risk for CAPA. Others authors [10, 11, 19] highlighted how renal replacement therapy was more frequent in CAPA patients and that CAPA diagnosis was associated with an increased need of renal replacement therapy. One patient in Van Biesen et al.’s [16] work was a kidney transplant recipient.

### ICU management factors

#### Time to diagnosis

Despite the objective difficulties in identifying the precise time of the first clinical suspicion and the actual development of aspergillosis, several studies reported the temporal gap between the onset of COVID-19 and aspergillosis [11, 15, 26, 28, 29, 32]. Median value derived from this cohort is 11 days.

It has also been reported that the diagnosis of aspergillosis is made, on average, 8 days after admission to intensive care [15, 26, 29, 32] and, on average, 6.8 days after intubation [25, 31].

The time elapsing between hospitalization and the finding of positive culture samples ranges between 8 and 19.5 days [30, 33].

It is interesting to note that there is evidence that appears to correlate the length of stay in the ICU with the development of aspergillosis (12 days in patients without aspergillosis, 23 days in those colonized and 27 days in those with presumed/probable invasive aspergillosis) [24].

#### Respiratory support

Data relating to the frequency of IPA in patients treated with mechanical ventilation in ICU are very heterogeneous (range 4–50%) [11, 15, 16, 19, 24, 25, 28, 32]. Furthermore, IPA diagnosis have also been described in patients treated only with non-invasive respiratory support (range 18–33%) [14, 26, 33].

#### COVID-19 immunomodulating therapies

Among the patients included in the present analysis, 53% received corticosteroid (88/166) with an average of 76.1 mg/day of prednisolone equivalent. Considering tocilizumab, patients treated in studies considered varied from 14.3 to 100% [29, 31–33].

Immunomodulating therapy was administered in 39 patients (data available in eight studies only). For the majority of patients, (34/39) anti IL-6 receptor tocilizumab was prescribed. No data on other immunomodulant therapies were reported.

A likely correlation between corticosteroids use and development of aspergillosis is hypothesized [11, 12, 26] even though the number of patients treated with steroids is very heterogeneous (from 11.1 to 77.8%).

#### Intercurrent infections

Among the included studies, none described the presence of Aspergillus infection in the context of other superinfections. Even if evidence of previous bacterial infections has been reported, no correlation with the development of aspergillosis has been proposed. Particularly informative about bacterial and fungal superinfection rates and antimicrobial treatment is the study by Rothe et al. [34] who, in a group of 50 critically ill patients admitted to the intensive care unit, described *Enterobacterales* (34.0%) and *Aspergillus fumigatus* (18.0%) co-infections.

#### Previous antibiotics and antifungal treatment

Although many studies have described the use of broad-spectrum antibiotic therapy, there is a lack of systematic data on antimicrobial therapies prior to Aspergillus infection. Evidence from this analysis suggests that patients who developed aspergillosis had previously been treated with broad-spectrum antibiotics [31], piperacillin/tazobactam and meropenem [35]. Evidence for patients treated with carbapenems is limited and inconclusive [34, 35].

#### Environmental and logistic factors

Analysing the impact of environmental and logistical factors, a role in facilitating the development of pulmonary aspergillosis in COVID-19 patients of negative atmospheric pressure in intensive care rooms was highlighted [36].

It is however suggested to exclude an environmental source by sampling room air, oxygen and pressurized air supplies and installing high efficiency particulate air filters [25].

## Discussion

Even if fungal superinfections are emerging as a key determinant of severity and mortality in critically ill COVID-19 patients, no study, to the best of our knowledge, focused on the host and risk factors for the development of aspergillosis in COVID-19, which is pivotal in guiding early suspicion and diagnostic approaches.

Among the host-related comorbidities, known traditional risk factors for aspergillosis, such as respiratory disease (notably COPD and asthma), have been confirmed to be associated with CAPA in ICU patients diagnosed with COVID-19 [14, 29, 37].

Cardiovascular disease, mainly arterial hypertension and coronary heart disease, are also frequently reported. However, as already pointed out by others, such an association seems to be more attributable to the prevalence of these conditions in the general population rather than a correlating factor with the severe form of COVID-19 [23, 28, 38].

Little is known about the impact of liver disease on the development and outcomes of CAPA, and to date, no data seems to indicate hepatic involvement as a clear risk factor for CAPA.

Although diabetes has been frequently observed in patients with COVID-19 and CAPA, it is not possible to draw conclusions from the results of our review, since, in the vast majority of reports, staging of the disease was not available and no causal relationship was either proposed or demonstrated.

Chronic kidney disease also appears to be quite common in CAPA patients. Since renal complications are known to arise mainly in patients with ARDS, there are no indications correlating these conditions to CAPA, while some data seems conflicting on that theme specially for patients undergoing RRT [11].

A significant number of potential risk factors for CAPA seem related to the overall management of critically ill patients in ICU and targeted therapies for COVID-19.

Although respiratory support—a cornerstone of COVID-19 supportive therapy—is recognized as a risk factor for IA IPA [39–41], there is no evidence evaluating the different impacts of invasive and non-invasive ventilation (NIV). Notwithstanding, a possible role of NIV in contributing to ventilator-induced lung injury, and hence to aspergillosis, cannot be ruled out, and its contribution to healthcare-associated or ventilator-associated pneumonia remains to be studied in more detail.

Undoubtedly, data referring to the time elapsed between COVID-19 and the diagnosis of CAPA are intriguing. It seems that time span is longer than the median time between influenza and IAPA diagnosis [42], as is the time between ICU admission and CAPA development [43]. Factors possibly explaining these differences include the different timing of diagnostic tests used in different countries and hospitals. Furthermore, some diagnostic results seem to derive from tests performed in the context of screening panels [11, 12, 24, 25], while others derive from tests requested on clinical indications [32, 35]. Finally, significant differences in diagnostic latency can derive from the use of different and non-standardized screening strategies such as fibrobronchoscopy, the measurement of galactomannan (GM), and the use of invasive broncho-alveolar lavage (BAL) in intubated patients.

It is difficult to correctly interpret the role played by steroid therapy given that, in many cases [14, 29, 31, 33], it has been administered in combination with tocilizumab and without detailed indications about the duration and doses used. In this case also it is likely that the various therapeutic approaches used in the months immediately following the development of the pandemic derive from a relative uncertainty about the effectiveness of this approach. This is particularly true in the context of this review that included studies published before the RECOVERY study [44] which standardized the use of steroids. Still unclear are even the role and possible side effects of interleukin inhibitors, although some evidence seems to suggest an impact favouring infections [45]. Finally, information on therapies other than tocilizumab (e.g., anti-interleukin 1) is missing.

The correlation between bacterial and viral superinfections and the development of CAPA in ICU COVID-19 patients has not been specifically investigated [19, 26, 34]. It is likely that the logistical difficulties associated with the pandemic spread have made the multi-resistant Gram-negative bacteria superinfections particularly frequent and severe [46, 47]. To date, only one study [34] specifically addresses the issue of the possible correlation between previous broad-spectrum antibiotic therapy (notably carbapenems) and fungal superinfections.

Finally, some additional considerations about CAPA can be drawn based on experiences with influenza-associated pulmonary aspergillosis (IAPA). Generally, compared to IAPA, the majority of CAPA cases have been classified as putative rather than proven/probable IPA in the absence of positive serum GM or histopathologic evidence. This largely reflects the pandemic situation in which diagnosis has been attempted. Different markers (PCR, GM, culture on specimen from bronchoscopic or non-bronchoscopic lavage) and different approaches to diagnosis may explain categorisation differences, as previously emerged for IAPA [48]. Comparison remains difficult because CAPA’s definition has long been unclear, although recently a consensus has been proposed [10]. Given the proposed case definition of IAPA, it is remarkable that it was observed among patients with ‘classic’ host factors predisposing them to IPA, which was not considered in the EORTC/MSGERC definition and AspICU algorithm. Accordingly, more similarities can be found between IAPA and CAPA, and the ‘entry criterion’ for both now are similar [10].

While it is clear that many uncertainties remain regarding CAPA’s definition and risk factors, it is nevertheless important to point that distinct physiopathology, cytokine profiles and therapies for influenza and COVID-19 may, at least partially, explain the observed discrepancies. Notably, the proportion of patients with classical immunosuppressive host factors appears to be higher among severe influenza compared to severe COVID-19 patients. Additionally, patients diagnosed with IAPA are younger and affected by a more severe clinical presentation than those diagnosed with CAPA, but mortality is similar [49]. ARDS in COVID-19 is predominantly observed among a specific category of patients with no particular risk for IPA, except for hypertension, diabetes mellitus and obesity, as outlined in this review. Similarly to what happens in influenza, lymphopenia is a common feature of severe COVID-19, but epithelial damage presents some differences, being extensive, with destruction and necrosis of the respiratory tracheobronchial epithelium in influenza, and milder, due to endothelial and cytokine-mediated damage in COVID-19. Involved cytokines seem to be different, with a more oriented IL-6, TNF, interferon and CXCL5 profile in COVID-19 and a more defined role for IL-6 and IL-10 in influenza [48].

There is no doubt that the role of other elements of COVID-19 pathobiology need to be explored and it is likely that the particular environmental factors characterizing this pandemic could play a wider role than what has been ascertained to date. Negative atmospheric pressure used in ICU rooms may represent a risk for pulmonary aspergillosis in COVID-19 patients [50] and the change in the ICU environment due to the COVID-19 pandemic may request specific interventions to rule out an environmental source of infection [25].

## Conclusion and proposal for future research area

Despite the growing interest in the role of invasive aspergillosis in COVID-19 patients, most of the existing evidence comes from case series, mostly retrospective, collected in European countries. The association between COVID-19 and Aspergillosis, underlying the new CAPA definition [48], still needs to be definitively demonstrated, as in the case of influenza [10].

Ongoing efforts for more standardized diagnostic approaches and IPA definitions in ICU should be continued and prospectively validated, as the significance of Aspergillus microbiological identification or GM positivity in BAL and non-BAL respiratory samples is difficult to assess. As a consequence, a “clinical” diagnosis is far from to be obtained, leaving intensivists facing critically ill COVID-19 patients in the limbo of a challenging definition (possible vs proven/probable vs putative IPA, or colonization).

It is, however, noteworthy to understand that new risks factors are emerging. The attention is shifting from patients’ related risk factors to factors characterizing the hospital and intensive care course which in turn are deeply influenced by specific features of COVID treatment itself. Prolonged invasive or non-invasive respiratory support, widely used in the treatment of COVID-19 patients, is undoubtedly one of the most interesting factors to be explored as well as particular attention should be paid to the impact of corticosteroids and / or immunobiological therapies on incidence and mortality of CAPAs and the relationship between endothelial alterations induced by COVID-19 [51] and the immune response [52].

It is certainly to be considered as factors related to the ICU setting such as isolation conditions, ventilation systems, building renovation works, the need to manage a large influx of COVID-19 patients, may play a role so far underestimated.

Finally, it would be desirable that the impact of antifungal treatment (intended as prophylaxis, pre-emptive strategies or target treatment), on the incidence and mortality of CAPA should be evaluated with prospective studies, in order to allow stratify the risk and guide the clinical decision of when and how to treat this particular “at risk” population.

## Data Availability

Not applicable.
